# Species Distribution, Characterization, and Antifungal Susceptibility Patterns of *Candida* Isolates Causing Oral and Vulvovaginal Candidiasis in Chile

**DOI:** 10.3390/antibiotics14070712

**Published:** 2025-07-16

**Authors:** Francisca Nahuelcura, Eduardo Álvarez Duarte

**Affiliations:** Mycology Unit, Microbiology and Mycology Program, Biomedical Sciences Institute, University of Chile, Av. Independencia 1027, Santiago 8380453, Chile

**Keywords:** oral candidiasis, vulvovaginal candidiasis, non-*albicans* species, *Candida africana*, *Candida dubliniensis*, *Candida albicans*

## Abstract

Background: Oral candidiasis (OC) and vulvovaginal candidiasis (VVC) are infections caused by species belonging to the genus *Candida*. In Chile, epidemiological studies on OC/VVC are scarce, leading to an overestimation of the prevalence of *C. albicans*. Additionally, awareness of the prevalence of species phenotypically and genotypically similar to *C. albicans* is lacking. The clinical impact of non-albicans species in cases of OC/VVC is also often underestimated. This study aims to determine the distribution of *Candida* species, their phenotypic and molecular characteristics, and their antifungal susceptibility patterns in incidents of oral and vulvovaginal candidiasis in Chile. Methods: A descriptive analysis was conducted on 101 isolates of *Candida* spp. obtained from OC/VVC cases. The identification of *Candida* species was performed using both phenotypic and molecular techniques. Antifungal susceptibility testing was carried out using the Sensititre YeastOne system. Results: Among the analyzed isolates, 89.1% were identified as *C. albicans*, while 10.9% were categorized as non-albicans species, including *C. dubliniensis*, *C. glabrata sensu stricto*, *C. bracarensis*, *C. tropicalis*, *C. lusitaniae*, and *C. parapsilosis sensu stricto*. The susceptibility pattern was predominantly susceptible, with only 10.9% of the total strains demonstrating resistance, and low antifungal activity in vitro was observed for Fluconazole, Voriconazole, and Posaconazole. Conclusions: The most prevalent species causing OC/VVC in Chile is *C. albicans*. This study also presents the first report of *C. lusitaniae* as a causal agent of VVC in the country. The identification of azole-resistant strains emphasizes the critical role of laboratory diagnosis in VVC cases, thereby preventing potential treatment failures. No resistance was observed in the strains associated with OC.

## 1. Introduction

Oral candidiasis (OC) and vulvovaginal candidiasis (VVC) are infections caused by yeasts of the genus *Candida*, which are common reasons for seeking medical consultation regarding oral and vaginal infections [1,2]. OC is a significant global health issue, with prevalence rates that vary based on both population and geographic region. In patients with diabetes, the prevalence of OC is notably high, ranging from 50.8% to 67%. Several studies indicate that approximately 60% of these patients are affected, with *Candida albicans* being the predominant species [3,4]. In contrast, the prevalence in the general population is lower yet still significant. For instance, a study in Brazil found that 28.7% of hospitalized patients presented with oral candidiasis, with *C. albicans* identified as the most common species, representing 84% of cases [5].

In the context of VVC, *C. albicans* is identified as the predominant species, representing 80–90% of isolated cases. The remaining cases of VVC are predominantly attributed to non-albicans species, with *C. glabrata* being the most frequently reported among them [6]. Non-albicans species have been consistently linked to cases of VVC exhibiting antifungal resistance [1]. Previous studies have highlighted an increasing prevalence of VVC instances caused by non-albicans species [7]. This emerging trend is due to multiple factors, including insufficient laboratory identification of the causal agents of VVC and the unregulated availability of antifungal agents, which promotes the selection of resistant strains [1]. Moreover, non-albicans species are commonly linked to cases of VVC characterized by antifungal resistance [1].

Similar species, such as *Candida africana* and *Candida dubliniensis*, have been discovered as etiological agents of OC and VVC. These species are often misidentified in laboratory settings due to conventional techniques that fail to adequately differentiate between them. Epidemiological research indicates that *C. africana* has a global distribution. It has frequently been isolated from vaginal samples and reported in VVC cases in regions such as Africa, Germany, Spain, and Italy [8,9,10]. The taxonomic classification of *C. africana* has been a subject of controversy, with some authors considering it a distinct species, while others regard it as a genetic variant of *C. albicans*, resulting in a lack of consensus. Nevertheless, *C. africana* is commonly accepted as a synonym of *C. albicans* [10]. On the other hand, *C. dubliniensis* is usually associated with cases of oral candidiasis but has also been isolated from different anatomical sites, including vaginal samples [10,11]. Currently, there is no reported data on *C. dubliniensis* in the context of OC and VVC in Chile. It is essential to emphasize that the lack of incidence data and epidemiological information regarding VVC can be partially attributed to its classification as a non-obligatory notification disease [7]. Consequently, there remains a significant gap in knowledge concerning the epidemiology, identification of etiological agents, and antifungal susceptibility profiles associated with cases of OC and VVC in Chile. The objective of this study is to address these critical questions systematically.

## 2. Results

### 2.1. Phenotypic Identification

**Figure 1:**
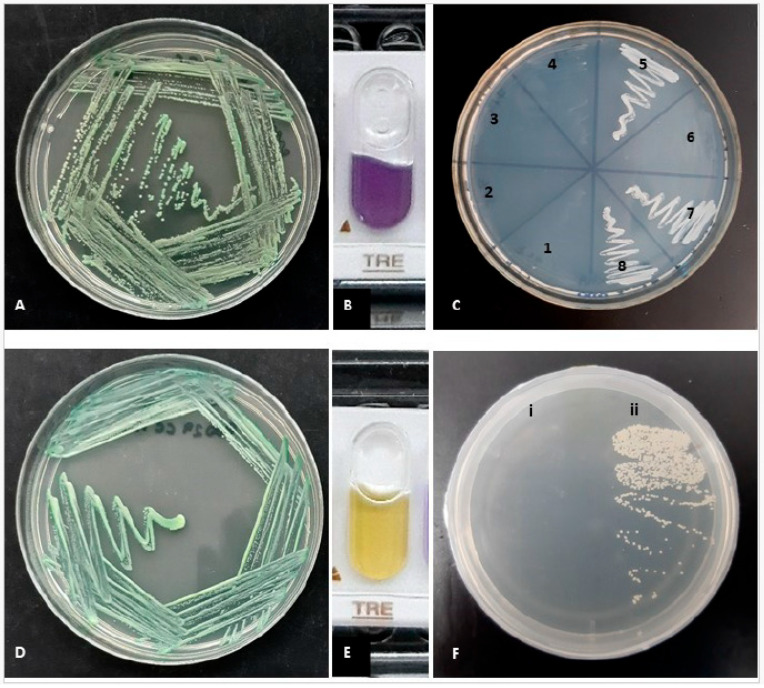
(**A**) *Candida dubliniensis* on CHROMO Candida. (**B**) *Candida dubliniensis* trehalose negative test, 24 h at 25 °C. (**C**) Xilose Hypertonic Agar, 72 h at 30 °C. 1 *C. dubliniensis* OC1010; 2 *C. dubliniensis* OC1040; 3 *C. dubliniensis* OC1050; 4 *C. dubliniensis* OC1060; 5 *C. albicans* 700; 6 *C. dubliniensis* OC1030; 7 *C. albicans* 612; 8 *C. albicans* OC3251. (**D**) *Candida albicans* on CHROMO Candida. (**E**) *Candida albicans* trehalose positive test, 24 h at 25 °C. (**F**) Xilose Hypertonic Agar, 72 h at 30 °C. (**i**) *C. dubliniensis* CD36; (**ii**) *C. albicans* ATCC 90028.

Among 101 strains cultured in CHROMagar CANDIDA, 95% (*n* = 96) of the strains exhibited green colonies within 24 h of incubation. Additionally, 1.98% produced lilac colonies (*n* = 2), 0.99% formed blue colonies (*n* = 1), and another 1.98% displayed white-pink colonies (*n* = 2). Among the strains that developed green colonies, six did not demonstrate turbidity in hypertonic broth after 96 h of incubation, indicating a negative result for this assay. Only one strain exhibited restricted growth at 42 °C after 48 h, while nine strains showed no growth at 45 °C. After 96 h of incubation at 30 °C, eight strains were unable to grow in the presence of xylose. Furthermore, on XHA agar, six strains failed to exhibit growth after 72 h of incubation. Quality control strains, specifically *C. albicans* ATCC 90028, ATCC 90029, and CECT 1001, yielded positive results across all four tests conducted. Notably, the type strain *C. dubliniensis* CD36 similarly showed no growth on XHA after 72 h of incubation. With API Candida, it was possible to identify most of the *Candida* strains with confidence. The strains belonging to *C. dubliniensis* could not be identified to the species level, since the gallery identifies them as *C. albicans*; however, as previously stated, the objective of using the galleries was basically to evaluate the sugar assimilation profile, given the easy interpretation of the API galleries. The trehalose test proved to be essential for distinguishing between *C. albicans* and *C. dubliniensis*. After 24 h of incubation at 37 °C, among the 96 strains that demonstrated green coloration on CHROMagar CANDIDA, six strains were found to be trehalose-negative, while the remaining 90 strains presented positive trehalose results. The six strains exhibiting a negative trehalose reaction were also the same strains that did not grow on XHA (Figure 1). These strains were subsequently identified as *Candida dubliniensis*, whereas the remaining 90 strains were classified as *C. albicans*.

### 2.2. Molecular Identification

According to the ITS region amplification of 101 *Candida* strains and the analysis carried out in BLASTn, it was obtained that the most prevalent species corresponds to *C. albicans*, which constituted 89.1% (*n* = 90) of the strains. Among these, 89.1% of the two strains, specifically ChFC 603 and ChFC 624, demonstrated a homology percentage of 98% with the sequence of *C. africana* (NR_138276.1) available in the NCBI database. Furthermore, this study presented non-albicans species associated with VVC, which accounted for 10.9% of the isolated strains. These included *C. glabrata sensu stricto* (*n* = 1), *C. bracarensis* (*n* = 1), *C. tropicalis* (*n* = 1), *C. lusitaniae* (*n* = 1), *C. parapsilosis sensu stricto* (*n* = 1), and *C. dubliniensis* (*n* = 6).

**Figure 2:**
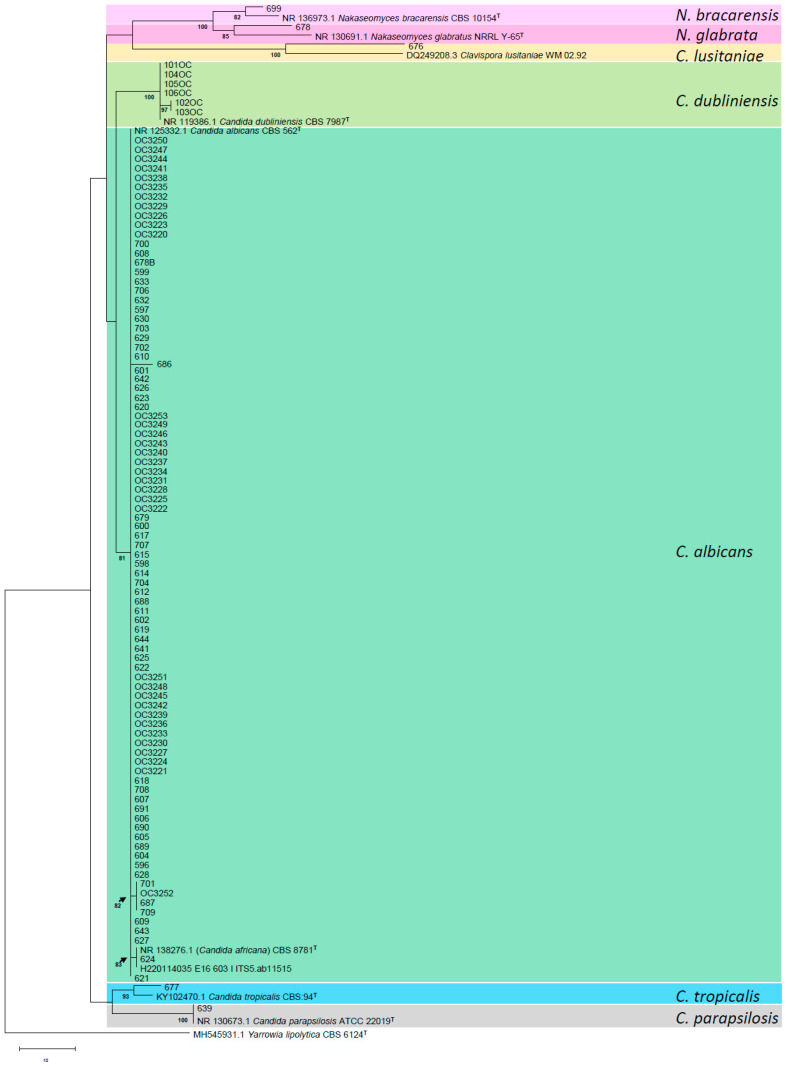
Maximum-likelihood (ML) tree obtained from ribosomal ITS regions and 5.8 S rRNA gene sequences of the strains analyzed in the present study, plus reference sequences obtained from the GenBank database. In the tree, branch lengths are proportional to distance. Bootstrap iteration frequencies (1000 iterations) above 70% are indicated on the nodes. Type or reference strains are indicated in boldface. T, type strain. Additionally, “OC” preceding the strain number indicates cases of oral candidiasis.

Phylogenetic analysis based on the ITS region indicated that the isolates were categorized into seven distinct clades: *albicans*, *dubliniensis*, *parapsilosis*, *tropicalis*, *lusitaniae*, *glabrata*, and *bracarensis* (Figure 2). The *albicans* clade includes *C. africana* CBS 8781, as well as strains ChFC 603 and 624, which exhibited a 98.86% identity with *C. africana.* A low percentage of genetic variability (0.6%) was observed among the *C. albicans* isolates, differing by three base pairs from their respective reference strain CBS 562. Additionally, the reference strain of *C. dubliniensis* CBS 7987 was positioned in a separate clade from its closely related species, which included six strains isolated from cases of oral candidiasis.

### 2.3. Susceptibility

Echinocandins, Amphotericin B, and 5-Fluorocytosine showed high activity against the 101 strains of *Candida* spp. presenting low minimum inhibitory concentrations (MICs) (MIC < 0.5 μg/mL) (Table 1).

Within the *C. albicans* isolates, azole MIC displayed variability. Five isolates demonstrated resistance (*n* = 3) or dose-dependent susceptibility (*n* = 2) to Fluconazole, with MIC values ranging from 4 to 16 μg/mL. These isolates also exhibited resistance (two isolates) or intermediate susceptibility (two isolates) to Voriconazole, with MICs of 1 μg/mL and 0.25 μg/mL, respectively. All five isolates were classified as non-wild-type strains concerning Posaconazole, with MICs between 0.25 and 0.5 μg/mL. However, the majority of the *C. albicans* isolates included in this study were deemed susceptible strains.

Regarding the *C. glabrata* complex, which includes *C. glabrata sensu stricto* and *C. bracarensis*, these strains demonstrated dose-dependent susceptibility to Fluconazole, with MICs ranging from 4 to 8 μg/mL, thereby categorizing them as wild strains for Fluconazole. *C. tropicalis* was found to be within the wild range and was susceptible to all azoles, with the exception of Posaconazole, which recorded an MIC of 0.25 μg/mL. Additionally, *C. lusitaniae* exhibited low MICs across all azoles, while the *C. parapsilosis* isolate presented similar characteristics.

## 3. Discussion

Approximately 1.5 million people worldwide are affected by *Candida* infections. Although bloodstream infections and invasive candidiasis are the most severe manifestations, oral candidiasis (OC) and vulvovaginal candidiasis (VVC) can induce considerable discomfort and complications, particularly when caused by antifungal-resistant strains. VVC, a vaginal infection, affects about 75% of women of childbearing age at least once in their lives [7,12]. The condition is predominantly induced by yeasts from the genus *Candida*, with *C. albicans* being the most prevalent species [7]. However, there has been a recent increase in VVC cases caused by non-albicans species, particularly *C. glabrata* [7,13]. In this study, 91.8% (*n* = 56) of the VVC strains analyzed were identified as *C. albicans*. Additionally, non-albicans species, accounting for 8.2% of the strains, included *C. glabrata sensu stricto*, *C. bracarensis*, *C. tropicalis*, *C. parapsilosis sensu stricto*, and *C. lusitaniae*. These species have all been previously identified as causative agents of VVC in international studies [7,13,14]. In Chile, Gática et al. [15] noted the isolation of *C. glabrata*, *C. tropicalis*, and *C. parapsilosis*; however, there have been no documented cases of *C. lusitaniae* as a causal agent of VVC in the country. International studies have indicated that *C. africana* has a higher incidence than *C. dubliniensis* among species closely related to *C. albicans* [8,16,17]. Furthermore, *C. dubliniensis* is more frequently associated with cases of oral candidiasis [10,11]. In the present study, six strains were identified as *C. dubliniensis*, constituting 5.9% of all *Candida* strains analyzed and 15% of those linked to oral candidiasis.

This study identified 15 strains suspected to be analogous to *C. albicans* based on phenotypic assessments. However, molecular analysis demonstrated that two of these strains had sequences with over 98% identity to sequences categorized as *C. africana* in the GenBank database. A phylogenetic analysis of the internal transcribed spacer (ITS) region revealed minimal genetic variability (0.6%) between these two strains and the reference strain of *C. albicans*, all of which were categorized within the same clade. Therefore, it is relevant to reassess the classification of *C. africana,* as it may not constitute a “similar species” to *C. albicans*, as is the case with *C. dubliniensis*, but rather a variant of *C. albicans*, as posited by other studies [10,18,19].

Phenotypic tests have revealed the occurrence of false positives, a problem confirmed by various authors using similar methodologies in their studies [20,21]. False positives can be attributed to atypical strains of *C. albicans* exhibiting these phenotypic traits [20], or they may represent genetic variants of *C. albicans* [10]. Recently, the term “*Candida albicans* species complex” has become prominent in Latin America, referring to *C. albicans*, *C. africana*, and *C. dubliniensis*, which suggests that these species are phenotypically indistinguishable yet distinct at the molecular level. This categorization may be inconsequential, however, as *C. africana* seems to be a genetic variant of *C. albicans*. Conversely, *C. dubliniensis* is sufficiently distinct to warrant its classification as a separate species owing to its notable molecular and phenotypic differences.

The results of the present study corroborate findings published by Jan et al. [22], in which none of the strains molecularly identified as *C. dubliniensis* demonstrated growth on XHA agar, unlike reference strains and clinical samples identified as *C. albicans*. Additionally, our results align with those of Kirkpatrick et al., who effectively identified *C. albicans* and *C. dubliniensis* strains through xylose assimilation in a cohort of oropharyngeal samples [23]. It is noteworthy that none of these six strains were able to utilize trehalose, a trait unique to *C. albicans* strains.

Given the findings mentioned above, it is advisable to avoid using the term “*Candida albicans* species complex,” particularly if it is intended to include *C. dubliniensis* inaccurately. This recommendation is especially crucial, considering that *C. dubliniensis* has been reported to rapidly acquire resistance to Fluconazole, leading to relapses or recurrences in patients undergoing traditional treatment regimens [24]. In terms of susceptibility profiles, Amphotericin B, 5-Fluorocytosine, and Echinocandins exhibit considerable efficacy against 101 strains of *Candida* spp., with minimum inhibitory concentrations (MICs) below 0.5 μg/mL. It is important to emphasize that Amphotericin B and 5-Fluorocytosine, when applied topically, are recognized as therapeutic alternatives for cases of vulvovaginal candidiasis (VVC) in instances of treatment failure with oral azoles. Such treatment failures are often associated with the isolation of non-albicans species, including *C. krusei* and *C. glabrata*, or strains of *C. albicans* that exhibit resistance to azole antifungals [25].

Strains of *C. albicans* have demonstrated resistance to Fluconazole, the primary antifungal treatment for this infection, particularly in cases involving azoles [7,26]. This resistance has been linked to therapeutic failures and recurrent vulvovaginal candidiasis (RVVC) [26]. In the present study, three *C. albicans* isolates were identified that exhibited resistance to Fluconazole (with MIC ranging from 8 to 16 μg/mL) and displayed either resistance or intermediate susceptibility to Voriconazole, while being classified as non-wild strains for Posaconazole. A similar finding was reported by Hong et al. [27], who isolated 23 strains showing cross-resistance to both Fluconazole and Voriconazole. Interestingly, our findings indicate that Itraconazole demonstrated significant activity not only against *C. albicans* strains but also against non-albicans species, with low MICs of 0.015–0.5 μg/mL. This stands in contrast to the study by Hong et al. [27], which found that 15.6% of *C. albicans* strains exhibited MICs above 0.5 μg/mL for Itraconazole.

Conversely, the species within the *C. glabrata* complex (including *C. glabrata sensu stricto* and *C. bracarensis*) showed low MIC values (4–8 μg/mL) interpreted as dose-dependent, with no resistant strains detected in this study—a finding consistent with reports by Gamarra et al. in Argentina [28].

For the remaining species, including *C. dubliniensis*, *C. parapsilosis sensu stricto*, *C. lusitaniae*, and *C. tropicalis*, low MICs were observed for all four azoles, except for *C. tropicalis*, which had a strain classified as the non-wild type for Posaconazole. Our results contrast with those reported by Hashemi et al. [14] and Mukasa et al. [29], who documented instances of *C. parapsilosis* strains causing VVC with resistance to Itraconazole and Fluconazole, respectively, and identified isolates of Itraconazole-resistant *C. lusitaniae*.

## 4. Materials and Methods

A total of 101 *Candida* species were isolated from cases of Oropharyngeal Candidiasis (OC) (*n* = 40) and Vulvovaginal Candidiasis (VVC) (*n* = 61). For the purpose of quality control, reference and type strains were utilized, including *C. albicans* ATCC 90028, ATCC 90029, CECT 1001, *C. dubliniensis* CD36, and *Candida parapsilosis* ATCC 22019.

### 4.1. Phenotypic Identification

The strains were cultured on SDA (Sabouraud Dextrose Agar with chloramphenicol) and CHROMagar CANDIDA (BD Difco™, Wokingham, Berkshire, UK) followed by incubation at 37 °C for a period of 24 to 48 h. The strains that exhibited green colony growth underwent a series of phenotypic tests to study species similar to *C. albicans*, including *C. dubliniensis* and *C. africana*. The hypertonic broth test was performed according to the guidelines established by Alves et al. [30]. The xylose-based agar medium was developed following the guidelines described by Khan et al. [13]. The capacity for growth at high temperatures was assessed at 42 °C and 45 °C. Additionally, the use of XHA (xylose hypertonic agar) medium was adopted to demonstrate the growth of the strains [22]. To objectively evaluate the utilization of carbon sources in a simple and reproducible manner, all *Candida* strains were tested using the API^®^ Candida system (bioMérieux, Marcy l’Etoile, France).

### 4.2. Molecular Identification

DNA extraction was conducted utilizing a lysis buffer and 0.5 mm diameter beads on the *Candida* spp. strains, which were incubated at a temperature of 37 °C on Sabouraud Dextrose Agar (SDA). The amplification of the internal transcribed spacer (ITS) region was executed using the primers ITS5 (forward primer: 5′-GGAAGTAAAAGTCGTAACAAGG-3′) and ITS4 (reverse primer: 5′-TCCTCCGCTTATTGATATGC-3′). The amplification products were subsequently purified with the PureLink™ Quick Gel Extraction Kit (Thermo Fisher Scientific, Waltham, MA, USA), in accordance with the manufacturer’s guidelines. Sequencing of the strains was performed at Macrogen Chile (Las Condes Santiago, Chile) using an ABI 3130 sequencer. Consensus sequences were generated employing the SeqMan software 7.0 (DNASTAR, Inc., Madison, WI, USA). The resulting DNA sequences were evaluated through the BLASTn search tool, referencing data from the GenBank database of the National Center for Biotechnology Information (NCBI). Multiple sequence alignment was accomplished using the MUSCLE program. For phylogenetic inferences, maximum-likelihood (ML) analysis was conducted using PhyML (version 3.0), applying heuristic search methods with starting trees derived from random addition with 100 replicates and nearest-neighbor interchange (NNI) branch swapping. For bootstrap ML analysis, we performed 1000 replicates (NJ starting tree with NNI branch swapping).

All the nucleotide sequences obtained in this study have been deposited in the GenBank database under the following accession numbers: PV800003-PV800102, PV837997.

### 4.3. Susceptibility

A susceptibility pattern was determined for 101 *Candida* strains using the Sensititre^®^ YeastOne^®^ Y010 AST Plate microdilution colorimetric method (Thermo Fisher Scientific, Waltham, MA, USA), in accordance with the manufacturer’s instructions for processing and interpretation. The *C. parapsilosis* ATCC 22019 strain was used as quality control. The interpretation of the results was made from clinical breakpoints (CBPs) of the M27M44S document of the Clinical and Laboratory Standards Institute (CLSI) [31]. In instances where CBPs were not available, the interpretation was guided by the epidemiological cutoff values (ECVs) outlined in the CLSI document M57 [32].

## 5. Conclusions

In conclusion, *C. albicans* remains the most frequently isolated species of OC/VVC in Chile. However, non-albicans species such as *C. dubliniensis*, *C. glabrata sensu stricto*, *C. bracarensis*, *C. tropicalis*, *C. parapsilosis sensu stricto*, and *C. lusitaniae* have been identified. Notably, this study presents the first documented instance of *C. lusitaniae* as a causative agent of VVC within the country. Furthermore, it is essential to emphasize the existence of the genotype “*C. africana*,” which has recently encountered challenges in its taxonomic classification. Through phylogenetic analysis of the ITS region, this study has classified it as part of the *C. albicans* clade, indicating that it should not be considered a distinct species but rather a genetic variant, in contrast to *C. dubliniensis*. Regarding phenotypic tests, this study revealed that certain isolates of *C. albicans* exhibited variable results in temperature and hypertonic broth tests, with some isolates demonstrating minimal or no growth. This phenomenon has been noted in previous research [20,21] and is thought to be associated with atypical strains or genetic variants of *C. albicans* [10]. Furthermore, the literature supports the utility of xylose and trehalose tests as effective methods for distinguishing between *C. albicans* and *C. dubliniensis*. Therefore, we strongly advocate for the use of the API Candida system, which has demonstrated its effectiveness as a rapid and objective method for trehalose analysis. The implementation of XHA agar has also proven successful in confirming the absence of growth in *C. dubliniensis* strains.

Given the emergence of azole-resistant strains, particularly those resistant to Fluconazole—the preferred treatment for VVC—it is essential to emphasize the importance of accurate laboratory diagnoses at the species level for VVC cases. Accordingly, we recommend that diagnostic protocols move towards specific identification rather than categorizing organisms as part of the “*C. albicans* species complex.” Additionally, it is crucial to conduct susceptibility profiling for all isolated agents, whether they are *C. albicans* or non-*albicans* species, to mitigate the risk of therapeutic failures.

To provide further clarity, additional studies are warranted to investigate the potential presence of resistance mechanisms or contributing factors in both resistant and non-wild strains assessed in this study. It is important to acknowledge that the availability of *C. dubliniensis* strains during our research was limited. Consequently, it is imperative to apply the aforementioned tests to a larger cohort of strains from this species. Such an approach will facilitate the validation of these tests, particularly for laboratories with lower complexity that lack access to advanced molecular biology techniques or MALDI-TOF technologies.

## Figures and Tables

**Table 1:** Results of the microdilution broth by Sensititre^®^ YeastOne Y010^®^ colorimetric methodology for the 101 strains of *Candida* spp. under study.

*Candida* spp.	Interpretation	AND	AMB	MCF	CAS	5-FC	POS	ITR	VRC	FLC
*C. albicans* (*n* = 90)	MIC range (μg/mL)	<0.015–0.12	0.12–0.25	<0.008–0.03	0.03–0.12	<0.06–0.5	<0.08–0.5	<0.015–0.5	<0.008–1	<0.12–16
	S/WT	90	90	90	90	90	85	90	86	85
	I/SDD	0	NA	0	0	NA	NA	NA	2	2
	R/non-WT	0	0	0	0	0	5 *	0	2 *	3 *
*C. dubliniensis* (*n* = 6)	MIC range (μg/mL)	<0.015–0.03	0.12–0.5	<0.008–0.03	0.03–0.06	<0.06–0.5	<0.08–0.12	<0.015–0.12	<0.008–0.06	<0.12–0.5
	S/WT	6	6	6	6	6	6	6	6	6
	I/SDD	0	NA	0	0	NA	NA	NA	0	0
	R/non-WT	0	0	0	0	0	0	0	0	0
*C. glabrata sensu stricto* (*n* = 1)	MIC range (μg/mL)	0.03	0.5	0.008	0.03	0.06	0.5	0.25	0.12	8
	S/WT	1	1	1	1	1	1	1	1	NA
	I/SDD	0	NA	0	0	NA	NA	NA	NA	1
	R/non-WT	0	0	0	0	0	0	0	0	0
*C. bracarensis* (*n* = 1)	MIC range (μg/mL)	0.12	0.12	0.015	0.06	0.06	0.25	0.12	0.06	4
	S/WT	1	1	1	1	1	1	1	1	NA
	I/SDD	0	NA	0	0	NA	NA	NA	NA	1
	R/non-WT	0	0	0	0	0	0	0	0	0
*C. tropicalis* (*n* = 1)	MIC range (μg/mL)	0.015	0.5	0.015	0.015	0.06	0.25	0.25	0.06	1
	S/WT	1	1	1	1	1	0	1	1	1
	I/SDD	0	NA	0	0	NA	NA	NA	0	0
	R/non-WT	0	0	0	0	0	1 *	0	0	0
*C. lusitaniae* (*n* = 1)	MIC range (μg/mL)	0.12	0.12	0.06	0.12	0.06	0.03	0.06	0.015	1
	S/WT	1	1	1	1	1	1	1	NA	1
	I/SDD	NA	NA	NA	NA	NA	NA	NA	NA	NA
	R/non-WT	0	0	0	0	0	0	0	NA	0
*C. parapsilosis sensu stricto* (*n* = 1)	MIC range (μg/mL)	0.5	0.25	1	0.5	0.06	0.03	0.06	0.008	0.5
	S/WT	1	1	1	1	1	1	1	1	1
	I/SDD	0	NA	0	0	NA	NA	NA	0	0
	R/non-WT	0	0	0	0	0	0	0	0	0

Abbreviations. MIC: minimum inhibitory concentration; AND: Anidulafungin; AMB: Amphotericin B. MCF: Micafungin; CAS: Caspofungin; 5-FC: 5-Fluorocytosine; POS: Posaconazole; ITR: Itraconazole; VRC: Voriconazole; FLC: Fluconazole; S: sensitive; WT: wild type; SDD: dose-dependent sensitive; I: intermediate sensitivity; R: resistant; non-WT: non-wild type; NA: not applicable; * R or non-WT from VVC samples.

## Data Availability

The data that support the findings of this study are available from the corresponding author upon reasonable request.
